# A disintegrin and metalloprotease 10 (ADAM10) is a central regulator of murine liver tissue homeostasis

**DOI:** 10.18632/oncotarget.7836

**Published:** 2016-03-01

**Authors:** Miryam Müller, Sebastian Wetzel, Julia Köhn-Gaone, Karel Chalupsky, Renate Lüllmann-Rauch, Roja Barikbin, Juri Bergmann, Birte Wöhner, Olga Zbodakova, Ivo Leuschner, Gregor Martin, Gisa Tiegs, Stefan Rose-John, Radislav Sedlacek, Janina E.E. Tirnitz-Parker, Paul Saftig, Dirk Schmidt-Arras

**Affiliations:** ^1^ Institute of Biochemistry, Christian-Albrechts-University, Kiel, Germany; ^2^ School of Biomedical Sciences, Curtin Health Innovation Research Institute, Faculty of Health Sciences, Curtin University, Bentley, Australia; ^3^ Laboratory of Transgenic Models of Diseases, Institute of Molecular Genetics of the ASCR, Prague, Czech Republic; ^4^ Institute of Anatomy, Christian-Albrechts-University, Kiel, Germany; ^5^ Institute of Experimental Immunology and Hepatology, University Medical Center Hamburg-Eppendorf, Hamburg, Germany; ^6^ Department of General and Thoracic Surgery, University Hospital Schleswig-Holstein, Campus Kiel, Kiel, Germany; ^7^ Institute of Pathology, University Hospital Schleswig-Holstein, Campus Kiel, Kiel, Germany; ^8^ School of Medicine and Pharmacology, University of Western Australia, Fremantle, Australia; ^9^ Laboratory of Integrative Biology, Institute of Molecular Genetics of the ASCR, Prague, Czech Republic

**Keywords:** ADAM10, liver progenitor cell, c-Met, Notch, hepatocyte differentiation, Pathology Section

## Abstract

A Disintegrin And Metalloprotease (ADAM) 10 exerts essential roles during organ development and tissue integrity in different organs, mainly through activation of the Notch pathway. However, only little is known about its implication in liver tissue physiology.

Here we show that in contrast to its role in other tissues, ADAM10 is dispensable for the Notch2-dependent biliary tree formation. However, we demonstrate that expression of bile acid transporters is dependent on ADAM10. Consequently, mice deficient for *Adam10* in hepatocytes, cholangiocytes and liver progenitor cells develop spontaneous hepatocyte necrosis and concomitant liver fibrosis.

We furthermore observed a strongly augmented ductular reaction in 15-week old ADAM10^Δhep/Δch^ mice and demonstrate that c-Met dependent liver progenitor cell activation is enhanced. Additionally, liver progenitor cells are primed to hepatocyte differentiation in the absence of ADAM10.

These findings show that ADAM10 is a novel central node controlling liver tissue homeostasis.

Highlights:

Loss of ADAM10 in murine liver results in hepatocyte necrosis and concomitant liver fibrosis.

ADAM10 directly regulates expression of bile acid transporters but is dispensable for Notch2-dependent formation of the biliary system.

Activation of liver progenitor cells is enhanced through increased c-Met signalling, in the absence of ADAM10.

Differentiation of liver progenitor cells to hepatocytes is augmented in the absence of ADAM10.

## INTRODUCTION

Proteolytic release of transmembrane ectodomains, also termed ectodomain shedding, is an irreversible post-translational mechanism to regulate protein function. Members of the A Disintegrin and Metalloprotease (ADAM) family are major mediators of receptor shedding. The family member ADAM10 has been identified as major sheddase for Notch receptors [1], thereby enabling Notch signalling. Complete knockout of *Adam10* in mice resulted in embryonic lethality phenocopying Notch1 knockout mice [1]. Conditional deletion of ADAM10 in intestinal [2] or haematopoietic [3] tissue clearly demonstrated its importance for progenitor cell-mediated tissue integrity. Strikingly, reduced progenitor cell proliferation in these mice was mainly linked to impaired Notch signalling [4].

In order to ensure tissue homeostasis under physiological stress conditions, the liver relies on its enormous regenerative capacity. Acute injury such as paracetamol poisoning or surgical removal of parts of the liver induce compensatory hypertrophy and proliferation of the remaining hepatocytes [5]. However, during chronic liver damage upon toxic insult or consistent viral load, hepatocyte proliferation is compromised due to replicative arrest. This leads to activation of stem cell-like liver progenitor cells (LPCs) that are capable of hepatocytic or cholangiocytic differentiation upon appropriate stimulation. After hepatocyte damage, phagocytosing macrophages secrete Wnt ligands which induce hepatocytic differentiation of LPCs. Biliary damage however, induces myofibroblasts to secrete Notch ligands in order to promote differentiation of LPCs into biliary epithelial cells [6]. The importance of the Notch pathway for liver development and liver regeneration has been further emphasised by mutations in the Notch ligand *Jagged-1,* as well as mutations in *Notch2* [7] which have been found in patients with Alagille syndrome. These patients develop cholestatic liver diseases due to bile duct paucity. In mice, deficiency for Notch2 resulted in impaired development of intrahepatic bile ducts accompanied by foci of hepatocyte death [8, 9].

In this study, we generated mice deficient for *Adam10* in α-fetoprotein (ALFP)-expressing bipotential hepatoblasts and consequently in adult LPCs, hepatocytes and cholangiocytes. Surprisingly, Notch2-dependent biliary tree formation was not impaired by the loss of ADAM10 activity. However, upon loss of hepatic ADAM10 we observed spontaneous hepatocyte necrosis due to downregulation of bile acid transporter expression, leading to a ductular reaction. We demonstrate that loss of ADAM10 in LPCs results in enhanced LPC proliferation by increased c-Met signalling, as well as in an augmented potential of LPCs to differentiate into hepatocytes. Taken together this study identifies ADAM10 as a central regulator of liver tissue homeostasis.

## RESULTS

### Hepatic deficiency of ADAM10 results in parenchymal liver damage and persistent liver fibrosis

ADAM10 is expressed in the liver and the extra-hepatic biliary system throughout embryonic development [10]. In order to analyse its physiological role in the liver we used Alfp-Cre transgenic mice [11] to limit ADAM10 deficiency to the liver. We confirmed exclusive Cre expression in adult hepatocytes and cholangiocytes by breeding Alfp-Cre mice to EYFP reporter mice (Figure S1A and S1B).

ADAM10^Δhep/Δch^ mice exhibited an efficient deletion of *Adam10* (Figure S1C) in the liver and a significant reduction of ADAM10 expression levels (Figure S1D). ADAM10 expression was not affected in other tissues (Figure S1E). ADAM10^Δhep/Δch^ mice were born at a mendelian ratio and normal vitality. ADAM10^Δhep/Δch^ mice were both generated on a pure C57BL/6 and a mixed 129/C57BL/6 background. Both mouse strains displayed a similar phenotype, supporting the notion that the observed phenotype developed due to a lack of ADAM10. We focused our analysis in the following mainly on ADAM10^Δhep/Δch^ mice on a mixed 129/C57BL/6 background as these mice developed the phenotype more rapidly.

**Figure 1:**
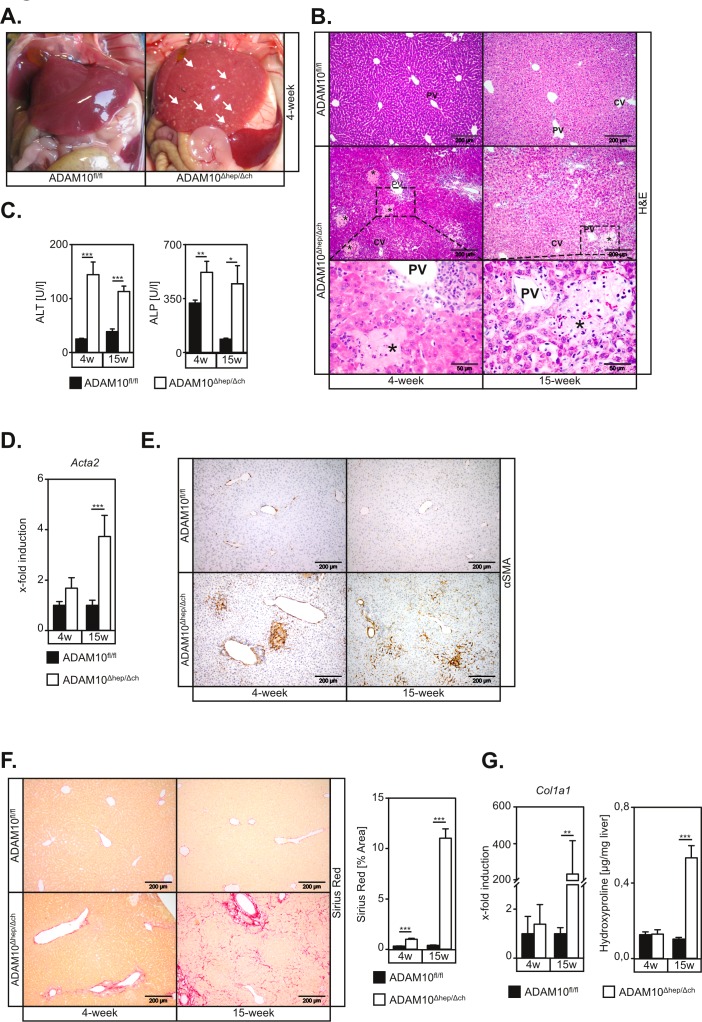
Mice with hepatic deficiency of ADAM10 develop spontaneous hepatocyte necrosis and concomitant liver fibrosis **A.** Necrotic lesions are macroscopically visible on livers of ADAM10^Δhep/Δch^ mice. White arrows indicate single necrotic lesions. **B.** H&E stainings of histological sections show necrotic areas predominantly in zone 2 of ADAM10-deficient livers. Necrotic areas are marked by asterisk. PV: portal vein; CV: central vein; Scale bars indicate 200 μm and in magnified areas 50 μm. **C.** ALT and ALP serum levels are upregulated in 4- and 15-week old ADAM10^Δhep/Δch^ mice compared to the respective controls (*n* = 3-16 animals for each subgroup). **D.** Expression of the αSMA gene (*Acta2*) is increased in livers of ADAM10^Δhep/Δch^ mice as assessed by qRT-PCR on mRNA from total liver tissue (*n* = 4-9 animals per subgroup). **E.** α-smooth muscle actin (αSMA) staining of histological liver sections reveals persistent activation of hepatic stellate cells in ADAM10-deficient livers. Scale bars indicate 200 μm. **F.** Sirius Red staining indicates increased liver fibrosis in ADAM10-deficient livers. Scale bars indicate 200 μm (*n* = 4-16 animals per subgroup). **G.** Quantification of hydroxyproline (*n* = 3-9 animals per subgroup) content and analysis of *Col1A1* expression (*n* = 4-9 animals per subgroup) in total liver extracts confirms increased liver fibrosis in ADAM10-deficient livers. Data represent the mean ± standard error of the mean. (* *P* < 0.005;** *P* < 0.01; ***P < 0.001).

Surprisingly, ADAM10^Δhep/Δch^ animals displayed macroscopic signs of liver parenchyma damage *in situ* (Figure 1A) accompanied by increased liver-to-body weight ratios in 4-week old animals (Figure S2A and S2B). ADAM10^Δhep/Δch^ mice revealed multiple foci of hepatocyte necrosis (Figure 1B and Figure S2C), predominantly located between the portal triad and the central vein (Figure 1B). Accordingly, we detected elevated serum levels of alanine transaminase (ALT) and alkaline phosphatase (ALP) in ADAM10^Δhep/Δch^ mice (Figure 1C and Figure S2D).

Hepatocyte damage was accompanied by a significant increase in hepatic stellate cell (HSC) activation, as evidenced by morphology (Figure S3A) and an increase of α smooth muscle actin (*Acta2*, αSMA) at the mRNA (Figure 1D) and the protein level (Figure 1E). Accordingly, we detected an upregulation of pro-fibrotic genes (Figure S3B-S3D) and liver fibrosis in 15-week old ADAM10^Δhep/Δch^ mice (Figure 1F and 1G, Figure S3E and F). All ADAM10^Δhep/Δch^ animals showed an increased spleen-to-body weight ratio (Figure S2B) which might be directly linked to liver fibrosis and a resulting portal hypertension.

### ADAM10 is dispensable for Notch-dependent biliary tree formation

**Figure 2:**
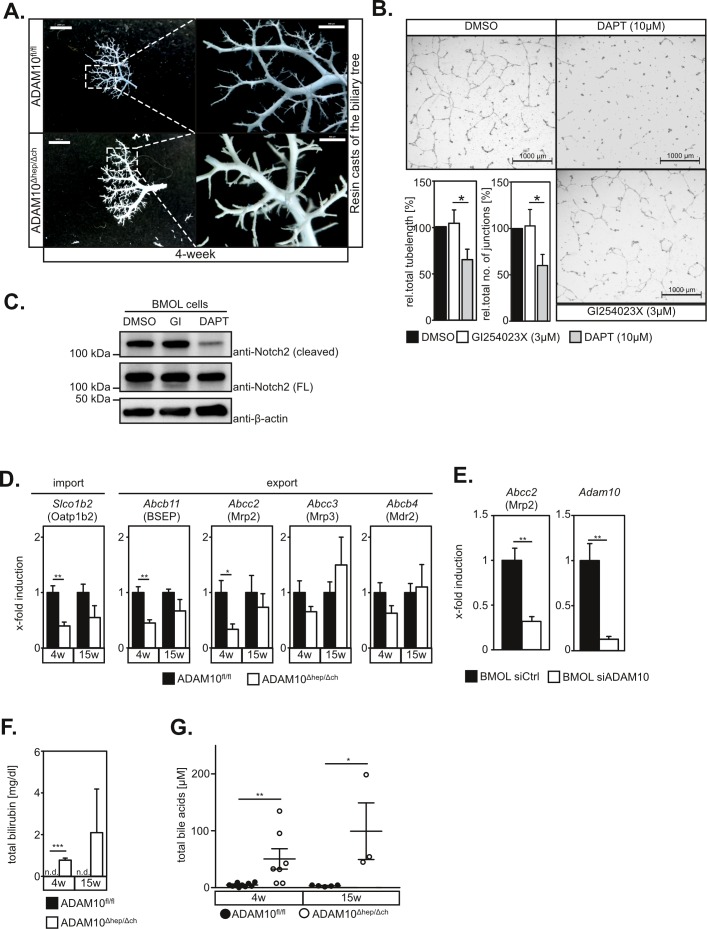
ADAM10 regulates expression of bile acid transporters but is dispensable for Notch-dependent biliary tree formation **A.** Resin casts of the biliary tree appear similar in 4-week old wildtype and ADAM10^Δhep/Δch^ mice. Scale bars indicate 2000 μm and in the magnified area 500 μm. **B.** Inhibition of ADAM10 does not affect tube formation of BMOL cells cultured on Matrigel. Total tube length and tubular junctions are significantly reduced in the presence of the γ-secretase inhibitor DAPT, but not in the presence of the ADAM10-inhibitor GI254023X. One representative and the quantification of five independent experiments (*n* = 5) are shown. Scale bars indicate 1000 μm. **C.** Notch2 cleavage in BMOL cells is not impaired by GI254023X-mediated inhibition of ADAM10 but abrogated upon DAPT-mediated γ-secretase inhibition. FL = full length **D.** Expression of *Abcb11*, *Slco1b2* and *Abcc2* genes, encoding for the bile acid transporters BCEP, Oatp1b2 and Mrp2 respectively, is downregulated in ADAM10^Δhep/Δch^ mice. (*n* = 4-5 animals for each subgroup). **E.** siRNA-mediated downregulation of ADAM10 in the murine LPC line BMOL results in transcriptional repression of the *Abcc2* gene encoding for Mrp2. (*n* = 4-6) **F.** Serum levels of total bilirubin are elevated in ADAM10^Δhep/Δch^ mice (*n* = 3 animals for each subgroup). **G.** Serum levels of total bile acids are elevated in ADAM10^Δhep/Δch^ mice (*n* = 3-10 animals for each subgroup). Data shown represent mean ± standard error of the mean. (*P < 0.05; **P < 0.01; ***P < 0.001).

Previous reports have shown that biliary tree formation depends on Notch2 activation [12]. Given the prominent role of ADAM10 in Notch activation in other tissues, hypothesised that hepatocyte necrosis was due to malformation of the biliary system. Surprisingly, we did neither detect alterations in the overall biliary tree structure as assessed by retrograde resin cast (Figure 2A) nor abnormalities in the ultrastructural morphology of bile canaliculi (Figure S4A). In line with these findings, Notch2 cleavage and *in vitro* tube formation of the murine bipotential liver progenitor cell line BMOL [13] on matrigel, mimicking biliary tree formation, was not reduced by the ADAM10 inhibitor GI254023X nor ADAM10-specific siRNA (Figure 2B and 2C and Figure S4B). However, the γ-secretase inhibitor DAPT impeded Notch2 cleavage and tube formation on matrigel significantly (Figure 2B and 2C) as previously described [14]. We furthermore did not detect significant alterations in the expression of the Notch target genes *Hes1* and *Hey1* (data not shown). These data demonstrate that Notch2-dependent differentiation of hepatoblasts and LPCs into cholangiocytes is still functional in the absence of ADAM10.

### ADAM10 regulates expression of hepatic bile acid transporters

Defects of bile acid transporters can cause hepatocyte death and are involved in the pathogenesis of several liver diseases including cholestasis, fatty liver disease and liver cancer [15]. We therefore determined expression of bile acid transporters in total liver extracts. We found a significant reduction in *Abcb11*, *Slco1b2* and *Abcc2* mRNA expression levels (Figure 2D). Accordingly, we detected partial loss of Mrp2, encoded by *Abcc2* in liver tissue sections of ADAM10^Δhep/Δch^ mice (Figure S4C). The expression levels of *Abcc2*/Mrp2 correlated significantly to the expression levels of *Adam10*, while ALT serum levels inversely correlated significantly to *Adam10* expression levels (Figure S4D). Reduced expression of *Abcc2* was directly linked to ADAM10 deletion, since siRNA-mediated suppression of ADAM10 in BMOL cells (Figure 2E), but not inhibition of ADAM10 protease activity (Figure S4E), markedly reduced *Abcc2* gene expression. Deficiencies in Mrp2, BSEP and Oatp1b2 in humans and mice result in increased serum levels of bilirubin [15]. Accordingly, we detected elevated serum levels of total bilirubin (Figure 2F) and total biliary acids in ADAM10^Δhep/Δch^ mice (Figure 2G and Figure S4F).

### Hepatic loss of ADAM10 induces a ductular reaction

**Figure 3:**
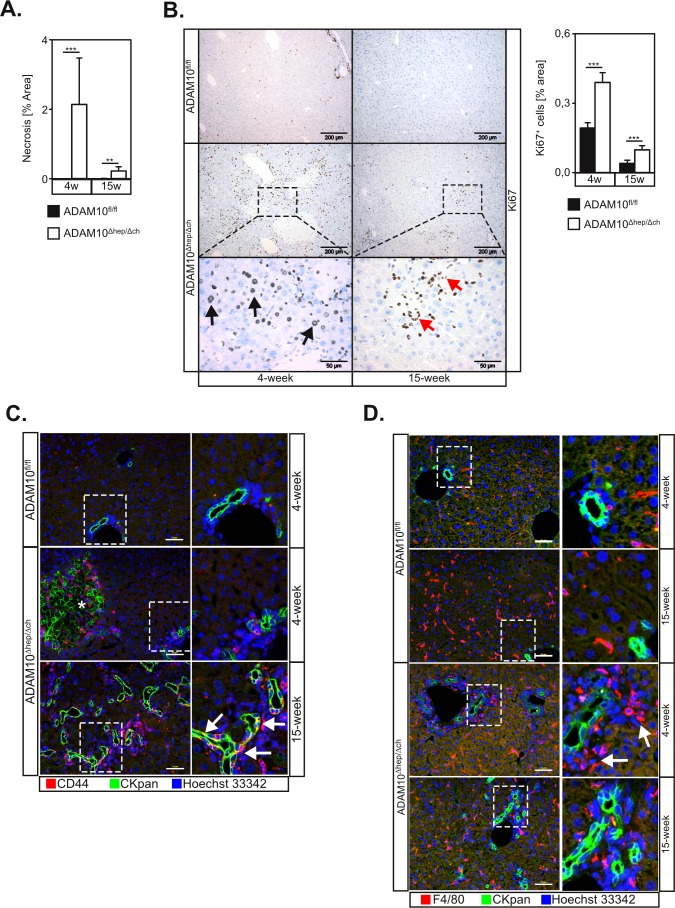
Loss of ADAM10 induces liver progenitor cell-mediated regeneration in ADAM10^Δhep/Δch^ animals **A.** Quantification of necrotic areas from histological sections (*n* = 4-9 animals for each subgroup) of ADAM10-deficient livers of 4- and 15-week old mice suggests a decrease of tissue damage over time. **B.** Increased proliferation in livers of ADAM10^Δhep/Δch^ mice as assessed by Ki67 staining. Scale bars indicate 200 μm and in the magnified area 50 μm. Black arrows designate Ki67^+^ hepatocytes. Red arrows indicate Ki67^+^ cells with a potential LPC morphology (*n* = 3-15 animals per subgroup). **C.** Ductular reaction and accumulation of LPCs in ADAM10^Δhep/Δch^ mice as marked by CKpan and CD44 staining of liver tissue sections of ADAM10^Δhep/Δch^ mice. Images from 4-week old wildtype animals are representative for both age groups. Asterisk mark unspecific CKpan staining of necrotic hepatocytes. Arrows indicate CKpan^+^ CD44^+^ LPCs. Scale bars indicate 50 μm. **D.** Clustering of macrophages around LPCs in 4-week old ADAM10^Δhep/Δch^ mice as marked by CKpan and F4/80 macrophage staining of liver tissue sections of ADAM10^Δhep/Δch^ mice. Arrows indicate clusters of F4/80^+^ macrophages. Scale bars indicate 50 μm. Data represent the mean ± standard error of the mean. (**P < 0.01; ***P < 0.001).

The area of liver damage was strongly reduced in 15-week old ADAM10^Δhep/Δch^ animals (Figure 3A), indicating an on-going regenerative process. We detected strong cell proliferation in liver tissue sections of ADAM10^Δhep/Δch^ mice, which was significantly increased compared to controls (Figure 3B). We detected Ki67^+^ nuclei in cells with hepatocytic morphology (Figure 3B, black arrows) but also in smaller epithelial cells, in particular in livers of 15-week old ADAM10^Δhep/Δch^ mice (Figure 3B, red arrows). The number of Ki67^+^ cells decreased in 15-week old ADAM10^Δhep/Δch^ animals but proliferation remained significantly upregulated compared to wildtype controls (Figure 3B).

We hypothesised that a subpopulation of small Ki67^+^ cells might be LPCs. We therefore analysed liver tissue sections by utilising antibodies against cytokeratin 19 (CK19) and pan-cytokeratin (CKpan) that stain biliary epithelial cells and LPCs. As expected, wildtype animals only showed CK19- and CKpan- positive staining in cholangiocytic cells in periportal regions. In 4-week old ADAM10^Δhep/Δch^ mice CK19^+^ and CKpan^+^ cells exclusively localised to portal ducts and necrotic areas that also stain due to cytokeratin rearrangement. In contrast, we detected an increase of CK19^+^ ductular structures with occasional single CK19^+^ and CKpan^+^ LPCs in the parenchyma in 15-week old ADAM10^Δhep/Δch^ mice (Figure 3C and Figure S5A). Importantly, at the later time point a population of CKpan^+^ cells also stained positive for CD44, indicating the presence of migratory stem-cell like liver progenitor cells (Figure 3C, white arrows).

Macrophage mobilisation has been observed to precede LPC activation [16]. Macrophages have been suggested as major regulators of LPC activation by producing the LPC mitogen TWEAK [17]. Interestingly, while the total number of F4/80^+^ macrophages was not increased in ADAM10^Δhep/Δch^ mice, F4/80^+^ macrophages underwent spatial reorganisation and accumulated around the ductular structures in ADAM10^Δhep/Δch^ mice compared to controls, suggesting an ongoing activation of LPCs (Figure 3D).

### LPC activation is enhanced in the absence of ADAM10

**Figure 4:**
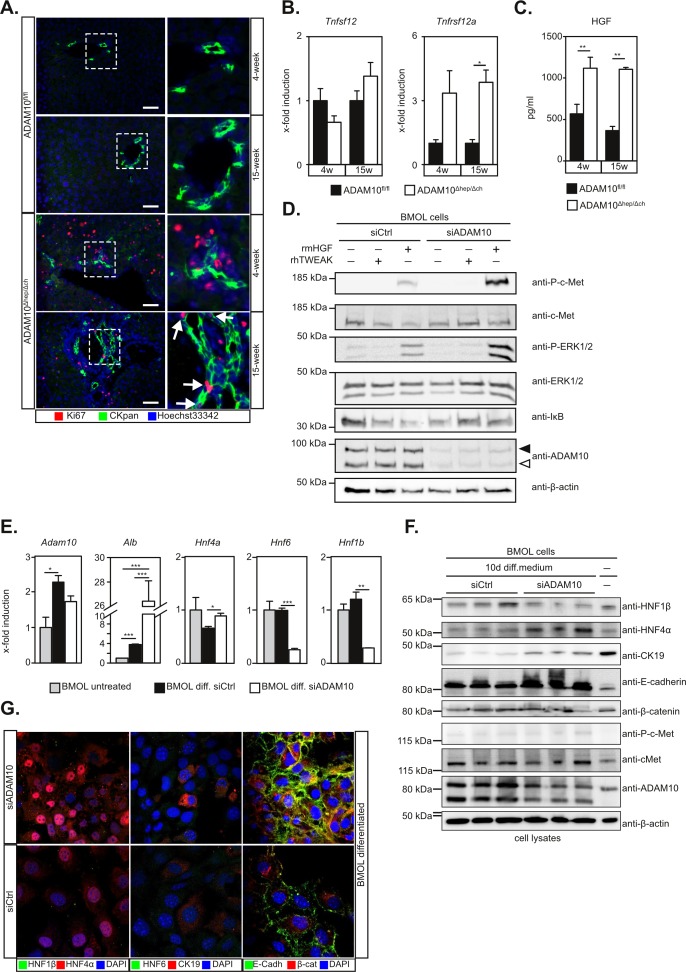
Activation of liver progenitor cells is enhanced in the absence of ADAM10 activity **A.** Detection of proliferating LPCs in 15-week old ADAM10^Δhep/Δch^ mice as assessed by CKpan/Ki67 immunofluorescent double staining. Arrows indicate CKpan^+^Ki67^+^ LPCs. Scale bars indicate 50 μm. **B.** Expression levels of *Tnfsf12* coding for TWEAK are unaltered while the expression levels of *Tnfrsf12a* coding for Fn14 are increased in ADAM10^Δhep/Δch^ mice (*n* = 4-5 animals per subgroup). **C.** Multiplex ELISA shows increased levels of the LPC mitogen HGF in the serum of ADAM10^Δhep/Δch^ mice (*n* = 3-15 animals per subgroup). **D.** siRNA-mediated downregulation of ADAM10 in BMOL cells results in enhanced HGF-induced c-Met signalling, but not TWEAK-induced Fn14 signalling. Filled arrowhead indicates ADAM10 pro-form. Open arrowhead indicates ADAM10 mature form. One representative out of three independent experiments is shown. **E.** Differentiation of BMOL cells to hepatocytes is enhanced in the absence of ADAM10 as assessed by qPCR analysis of hepatocytic genes *Alb* and *HNF4a* and cholangiocytic genes *HNF6* and *HNF1b*. **F.** Expression of HNF4α and the epithelial cell marker E-cadherin after differentiation is enhanced and expression of the cholangiocyte marker HNF1β is reduced in the absence of ADAM10 as assessed by immunoblotting. **G.** Immunofluorescent staining of siRNA-treated BMOL cells after differentiation reveals plasma membrane staining of E-cadherin, perinuclear staining of β-catenin and enhanced nuclear localisation of HNF4α in the absence of ADAM10. Data represent the mean ± standard error of the mean. (*P < 0.005; **P < 0.001, ***P < 0.001).

Immunofluorescent staining revealed proliferating CKpan^+^Ki67^+^ LPCs in 15-week old ADAM10^Δhep/Δch^ mice which were absent in 4-week old ADAM10^Δhep/Δch^ animals and wildtype controls (Figure 4A).

In accordance with previous reports [17] TWEAK (*Tnfsf12*) expression levels remained unchanged (Figure 4B). Transcript levels of the TWEAK-receptor Fn14 (*Tnfrsf12a*) were strongly upregulated in ADAM10^Δhep/Δch^ mice (Figure 4B), reflecting an increased number of LPCs in these animals.

HGF is upregulated and essential in mouse models of LPC-mediated regeneration [16]. We found a significant increase in serum levels of HGF in ADAM10^Δhep/Δch^ mice (Figure 4C). The receptor for HGF, c-Met, has been previously identified as a substrate of ADAM10-mediated shedding [18]. Both, siRNA-mediated ADAM10 suppression (Figure 4D) or pharmacological inhibition with GI254023X (Figure S5B) led to an enhanced c-Met and ERK1/2 phosphorylation in HGF-stimulated BMOL cells. We did not find a significant alteration of TWEAK signalling in BMOL cells in the absence of ADAM10, as evidenced by IκB degradation (Figure 4D and Figure S5B). These data indicate that enhanced pro-mitogenic-signalling in LPCs in the absence of ADAM10 activity is mediated by HGF.

### Hepatic differentiation of LPCs is enhanced in the absence of ADAM10

Previous reports have shown that during chronic liver damage LPCs can differentiate into hepatocytes [6]. As we observed ongoing regeneration in our ADAM10^Δhep/Δch^ mice, we adressed the potential of LPCs to differentiate into hepatocytes in the absence of ADAM10. We therefore subjected BMOL cells with siRNA-mediated ADAM10 suppression to previously described differentiation conditions (Figure S5C) [13]. ADAM10 expression was upregulated during differentiation in both control and ADAM10 siRNA-treated BMOL cells. Interestingly, expression of the cholangiocytic markers HNF6 and HNF1β were reduced significantly more in ADAM10-deficient BMOL cells on mRNA and protein level (Figure 4E-4G). Conversely, expression of the hepatocyte markers HNF4α and albumin, as well as the epithelial cell marker E-cadherin was markedly increased in ADAM10-suppressed BMOL cells as compared to control cells (Figure 4E-4G). Nuclear localisation of HNF4α was enhanced in the absence of ADAM10 (Figure 4G). We did not detect enhanced nuclear localisation of β-catenin, indicating that Wnt signalling was not involved under the conditions of differentiation used (Figure 4G). These data show that the potential of LPCs to differentiate to hepatocytes is enhanced in the absence of ADAM10.

## DISCUSSION

A Disintegrin And Metalloprotease (ADAM) 10 exerts essential roles during organ development and tissue integrity in many organs. In the present study we analysed its role in hepatocytes, cholangiocytes and liver progenitor cells using conditional *Adam10*-deficient mice.

In our study we found that ADAM10^Δhep/Δch^ mice suffered from hepatocyte necrosis and concomitant liver fibrosis. Previous reports have shown that in the liver, biliary tree formation is dependent on Notch2 activation [12]. Notch activation upon ligand-binding involves two consecutive proteolytic events leading to the generation of Notch intracellular domain and subsequent transcriptional regulation of Notch-target genes. Previous reports have demonstrated that in most tissues studied so far ADAM10 is responsible for the proteolytic cleavage of the Notch ectodomain [4].

Interestingly, ADAM10^Δhep/Δch^ mice did not display abnormalities in bile duct architecture or bile canaliculi morphology. Furthermore, *in vitro* tubulogenesis was also independent of ADAM10. Our data suggest that Notch2-dependent differentiation of hepatoblasts and bipotential liver progenitor cells into cholangiocytes is still functional in the absence of ADAM10 and is mediated by another protease. This is contrary to what has been observed before in other tissues, where loss of ADAM10 in the respective tissue stem cells impeded Notch-dependent stem cell activation [2, 3].

We observed down-regulation of the bile acid transporters Mrp2, BSEP, and Oatp1b2 in our mice and a concomitant increase in serum bilirubin, total bile acids, ALP and ALT. Deficiencies in Mrp2, BSEP and Oatp1b2 are linked to hyperbilirubinemia and cholestasis [15, 19]. We therefore hypothesise that in our mice loss of bile acid transporters results in impaired secretion of bile acids leading to hepatocyte necrosis. Loss of Mrp2 was directly linked to ADAM10-deficiency as corroborated *in vitro* in the murine bipotential LPC line BMOL. Previous studies showed that the intracellular domain (ICD) of ADAM10 can translocate to the nucleus [20], suggesting that ADAM10 ICD contributes to transcriptional regulation. Interestingly, in our experiments inhibition of ADAM10 activity was not sufficient to downregulate *Abcc2* expression in BMOL cells, suggesting that indeed ADAM10 ICD might be involved in the transcriptional regulation of *Abcc2* and other bile acid transporters.

Expansion of liver progenitor cells has been observed in a variety of human liver diseases such as chronical viral hepatitis, alcoholic liver disease and nonalcoholic fatty liver disease [21, 22]. Most likely as a consequence of persistent hepatocyte necrosis, ADAM10^Δhep/Δch^ mice developed a ductular reaction with activation of the CKpan^+^ LPC compartment. Interestingly, LPC proliferation persisted in ADAM10^Δhep/Δch^ mice at the later time point as marked by Ki67 staining despite a reduction in hepatic necrosis. We observed increased HGF signalling in the BMOL LPC line in the absence of ADAM10. ADAM10 therefore is an intrinsic negative regulator of LPC activation most likely through c-Met ectodomain shedding as reported to occur in other cell types [18].

Previous reports suggested that bipotential progenitors can emerge from adult hepatocytes through Wnt, Notch and TGF-β signalling [23-26]. At present we cannot exclude the possibility that accumulation of LPCs in ADAM10^Δhep/Δch^ mice is not only linked to enhanced LPC activation but may also be the result of de-differentiation of hepatocytes.

We furthermore found that LPCs had an increased potential to differentiate into hepatocytes in the absence of ADAM10. Under the differentiation conditions used we did not detect enhanced c-Met or Wnt signalling and the underlying molecular mechanism still has to be elucidaded. Interestingly, tissue inhibitor of metalloprotease 1 (TIMP-1), an inhibitor of ADAM10 [27], is strongly upregulated in chronic liver disease and might enhance LPC differentiation to hepatocytes through ADAM10 inhibition.

Taken together, we identified ADAM10 as a central node controlling liver tissue homeostasis based on three major findings: (I) ADAM10 or ADAM10-mediated signalling processes positively regulate expression of the bile acid transporters Mrp2, BSEP and Oatp1b2. (II) ADAM10 negatively regulates activation of liver progenitor cells through inactivation of c-Met signalling. (III) the potential of LPCs to differentiate into hepatocytes is augmented in the absence of ADAM10.

## MATERIALS AND METHODS

### Animal experimentation

To generate a hepatocyte- and cholangiocyte-specific knockout of ADAM10, homozygously floxed mice (ADAM10^fl/fl^, [1]) were crossed with Alfp-Cre transgenic mice (B6.Tg(Alb1-cre)7Gsc, [11]) and their offspring who carry homozygously floxed ADAM10 and are positive for Alfp-Cre were termed ADAM10^Δhep/Δch^. For additional information see Supplementary Experimental Procedures.

### Determination of alanine transaminase, alkaline phosphatase and bilirubin levels

Liver damage was assessed by measurement of ALT and ALP enzymatic activity and bilirubin levels in peripheral blood serum, according to the manufacturer's instructions (Reflotron^®^ Plus; Roche, Penzberg, Germany).

### Bile duct plastination

Plastination of the biliary tree was performed as described previously [9]. Casts were imaged using an AZ100 microscope with a DS-Fi2 camera (Nikon, Düsseldorf, Germany).

### Histological analysis and molecular techniques

See Supplementary Experimental Procedures

### Cell culture, treatments and functional studies

See Supplementary Experimental Procedures

### Statistics

Data were expressed as mean ± standard error of the mean. Comparisons between two groups were performed by applying the Student's t test. If data were not normally distributed or if they had unequal variances, the Mann-Whitney U test was employed for comparison of two groups. Measurement of the strength of the association between expressed genes or analytes was determined using Pearson product-moment correlation. All analyses were conducted using SigmaPlot 12.0 Software (Systat Software). A *p* value of < 0.05 was considered statistically significant.

## SUPPLEMENTARY MATERIAL FIGURES AND TABLES


