# Supplementary material for: A disintegrin and metalloprotease 10 (ADAM10) is a central regulator of murine liver tissue homeostasis
Source: Oncotarget. 2016 Mar 1;7(14):17431–41. doi: 10.18632/oncotarget.7836 (PMC4951223; doi:10.18632/oncotarget.7836)
Supplement: Supplementary file 1 [file oncotarget-07-17431-s001.pdf]

A disintegrin and metalloprotease 10 (ADAM10) is a central regulator of murine liver tissue homeostasis

Supplementary Material

Table of contents

1. Supplementary Experimental Procedure S1
2. Supplementary Figures S6
3. Supplementary Tables S14
4. Supplementary References S16

1. Supplementary Experimental Procedures

Animal experimentation

In this study we focused on 129/C57BL6 ADAM10$^{\Delta hep/\Delta ch}$ mice with a pronounced phenotype, which reliably appeared in 30-50% of the offspring. In some supplementary experiments, ADAM10$^{fl/fl}$ mice were crossed with Alb-Cre transgenic mice (B6.Cg-Tg(Alb-Cre)21Mgn [24]). The resulting litters used in these experiments were of a C57BL/6 background and homozygous for Alb-Cre and termed B6.ADAM10$^{\Delta hep/\Delta ch}$. In all experiments Cre-negative littermates served as controls unless stated otherwise. Heterozygous ROSA26-STOP-EYFP reporter mice (Jackson Laboratory, Stock# 006148) were used to examine Cre-induced recombination events. Both, male and female mice were included in this study and were analysed at the age of 4- or 15-weeks. All mice were housed under controlled conditions (Specific Pathogen Free, 22°C, 12-hour day-night cycle) and fed a standard laboratory chow *ad libitum*. Animals received humane care according to the criteria outlined by the governments of Germany and the Czech Republic. Detection of the recombined *Adam10* allele was performed by conventional PCR (~700 bp) on whole liver genomic DNA and subsequent agarose gel electrophoresis. Primer sequences are detailed in Supplementary Table 1.

Histological analysis

Liver tissues were fixed in phosphate buffered saline (PBS) containing 4% formaldehyde, dehydrated and embedded in paraffin, and cut into 4 µm-thick sections. Sections were stained with haematoxylin-eosin or Sirius Red using standard protocols. Additional sections were stained immunohistochemically. Primary antibodies were detected by biotinylated secondary antibodies and subsequently stained using a peroxidase DAB kit (Dako, Hamburg, Germany). Antibody specifications are listed in Supplementary Table 2. Haematoxylin was used for counterstaining. Necrotic, collagen containing and $Ki67^+$ areas were quantified by morphometry (ImageJ, NIH). For morphometric assessment, 10 non-overlapping fields at 10x magnification (necrosis) or 20x magnification (collagen, Ki67) per section were evaluated for each mouse. For immunofluorescence analyses, sections were deparaffinised, rehydrated and subjected to Proteinase K-mediated enzymatic or heat-induced antigen retrieval in either citrate buffer (pH6) or EDTA buffer (pH9). Sections were incubated with primary antibodies (Supplementary Table 2) overnight at 4°C and detected with Alexa Fluor® 488 or Alexa Fluor® 594 labeled secondary antibodies (1:200, Life Technologies, Victoria, Australia) for 1 hour at room temperature, all in Real™ antibody diluent (Dako, Hamburg, Germany). After counterstaining with Hoechst 33342, slides were mounted with ProLong® Antifade Reagent (Life Technologies, Victoria, Australia).

Immunofluorescence

Cells were grown on cover slips and fixed in 4% paraformaldehyde in PBS. Cells were permeabilized in 0.2% saponin in PBS and quenched in 0.12% glycin and 0.2% saponin in PBS. Cells were incubated with primary antibodies (Supplementary Table 2) overnight at 4°C and detected with Alexa Fluor® 488 or Alexa Fluor® 594 labeled secondary antibodies (1:200, Life Technologies, Darmstadt, Germany) for 1 hour at room temperature.

Coverslips were mounted with ProLong® Antifade Reagent with DAPI (Life Technologies, Darmstadt, Germany). Cells were then analysed on an Olympus FluoView 1000 confocal microscope.

Determination of total biliary acids

Total biliary acids from serum were determined using the total bile acids assay kit (Diazyme, Dresden, Germany) according to manufacturer's instructions. Absorption was measured at 405 nm in a microplate reader (Tecan, Mainz, Germany) or an AU480 (Beckman-Coulter, Germany) biochemical analyser.

BMOL tube formation assay

A 24-well plate was coated with 300 µl of slowly thawed Matrigel (Corning, Bedford (MA), USA) per well. Matrigel had a protein concentration of > 10 mg/ml and was left for at least 3h at 37°C, 5% CO_2, and 95% RH to solidify. 300 µl of a $2x10^5$ cells/ml cell suspension plus the respective inhibitors were seeded on top of the Matrigel in each pre-coated well. Cells were incubated for 24h to form tubes. Pictures of formed tubes were taken with a AZ100 microscope with a DS-Fi2 camera (Nikon, Düsseldorf, Germany) and tubelength and branching was evaluated with the ImageJ plugin 2D Skeleton.

BMOL cell stimulation

BMOL cells were kept under previously specified conditions (Tirnitz-Parker et al., 2007). For siRNA experiments 2.5×10^5 BMOL cells were seeded and transfected with 50 pmol control or mADAM10 siRNA (Life Technologies, Darmstadt, Germany), respectively. Transfection was performed at 0 h and again at 48 h after seeding using Interferin as transfection reagent (Hiss Diagnostics, Freiburg, Germany). Cells were used for stimulation experiments 72 h after seeding.

For stimulation experiments, BMOL cells were starved (0.5% FBS, 1mM Glutamine, 7.5 ng/ml IGF-II, 5 ng/ml EGF, 2.5 µg/ml Insulin) overnight and if indicated, pretreated with 3 µM ADAM10-Inhibitor GI254023X for 1 h prior to stimulation with 20 ng/ml rmHGF or 50 ng/ml rhTWEAK (Immunotools, Friesoythe, Germany) for 15 min at 37°C.

BMOL cell differentiation

2.5×10^5 BMOL cells were seeded either in six well plates or on cover slips and transfected with 50 pmol control or mADAM10 siRNA (Life Technologies, Darmstadt, Germany), respectively. Transfection was performed on day -4 and -2 and day 5 of differentiation (Figure S5C) using Interferin as transfection reagent (Hiss Diagnostics, Freiburg, Germany). Four days after intital cell seeding, medium was changed to differentiation medium as described previously (Tirnitz-Parker et al., 2007), which was replaced again on day 3, 5 and 7 (Figure S5C). Cells were harvested on day 10 of differentiation and either subjected to lysis, RNA isolation or immunofluorescence staining.

Protein isolation and immunoblotting

Samples were lysed in RIPA buffer supplied with 50 mM NaF, protease and phosphatase inhibitors. Proteins were separated by electrophoresis on 10 % SDS gels and transferred to PVDF or nitrocellulose membranes. Membranes were incubated with primary antibodies (Supplementary Table 2) overnight at 4°C and with horseradish peroxidase-conjugated secondary antibodies at room temperature for 1h. An ECL substrate kit was used for detection (Thermo Scientific, St. Leon-Rot, Germany).

Gene expression analysis

Total RNA was isolated from cryopreserved whole liver tissue using TRIzol (LifeTechnologies, Darmstadt, Germany) according to the manufacturer's instructions. One microgram of total RNA was used for reverse transcription with oligo-$(dT)_{18}$ primers and RevertAid Reverse Transcriptase (Thermo Scientific, St. Leon-Rot, Germany) and subsequently subjected to quantitative polymerase chain reaction. Respective gene expression levels were normalised to the expression of β-actin or tubulin and expressed as fold changes relative to normalised levels in the corresponding control mice. Primer sequences are detailed in Supplementary Table 1.

Hydroxyproline assay

The assay was performed as described previously (Uchinami et al., 2006).

Multiplex ELISA

Levels of HGF were measured by multiplex assay with a Bio-Plex mouse array (Bio-Rad Laboratories, Prague, Czech Republic) using the Bio-Plex 200 System (Bio-Rad Laboratories, Prague, Czech Republic). Mouse HGF multiplex beads were produced by amine coupling reaction with primary antibody from R&D (Minneapolis, USA) and validated according to Luminex validation procedures and recommendations.

Transmission electron microscopy

Liver tissue was fixed by immersion in 6% glutaraldehyde in 0.1 M phosphate buffer pH 7.2, postfixed with 2% osmium tetroxide and embedded in araldite. Ultrathin sections were stained with uranyl acetate and lead citrate and imaged with an EM900 Zeiss electron microscope.

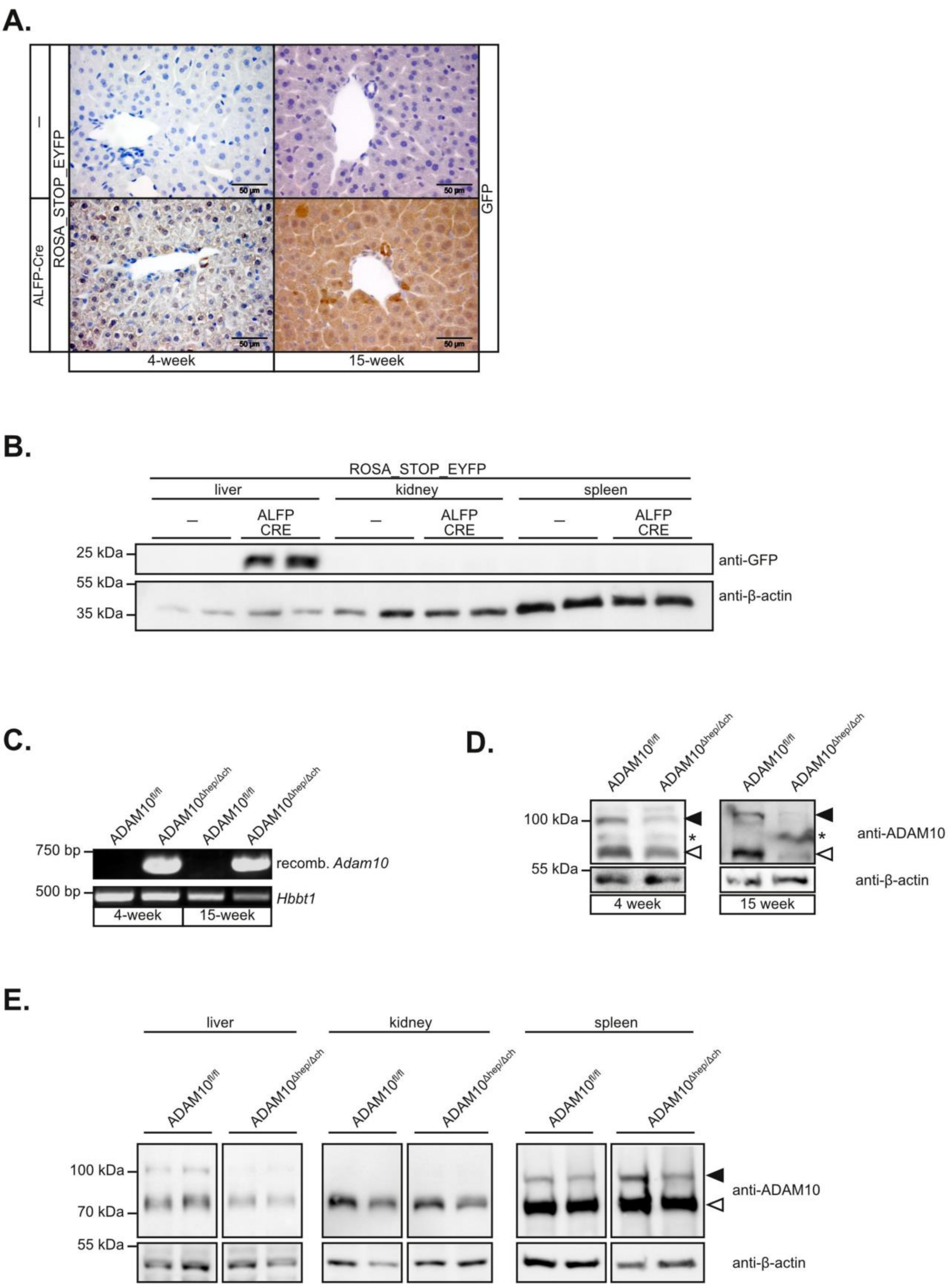

Supplementary Figure 1. A.+B. Alfp-Cre targets hepatocytes and cholangiocytes as demonstrated by cross-breeding with ROSA-STOP-EYFP mice and **(A.)** subsequent anti-

GFP immunohistochemistry of liver tissue sections and **(B.)** immunoblotting of total liver, kidney and spleen extracts using anti-GFP antibodies. Scale bars indicate 50 µm. **C.** Successful recombination of *Adam10* Exon 2 in ADAM10$^{fl/fl}$ mice expressing Cre under the *Alb* promotor and *Afp* enhancer elements as revealed by recombination-specific PCR. **D.** ADAM10 protein expression is decreased in total liver extracts of ADAM10$^{fl/fl}$ mice expressing Cre under the *Alb* promotor and *Afp* enhancer elements. Filled arrowhead indicates ADAM10 pro-form. Open arrowhead indicates ADAM10 mature form. Asterisks mark unspecific band. **E.** Alfp-Cre does not target epithelial cells in other organs as shown by anti-ADAM10 immunoblotting of total tissue extracts of liver, kidney and spleen. Filled arrowhead indicates ADAM10 pro-form. Open arrowhead indicates ADAM10 mature form.

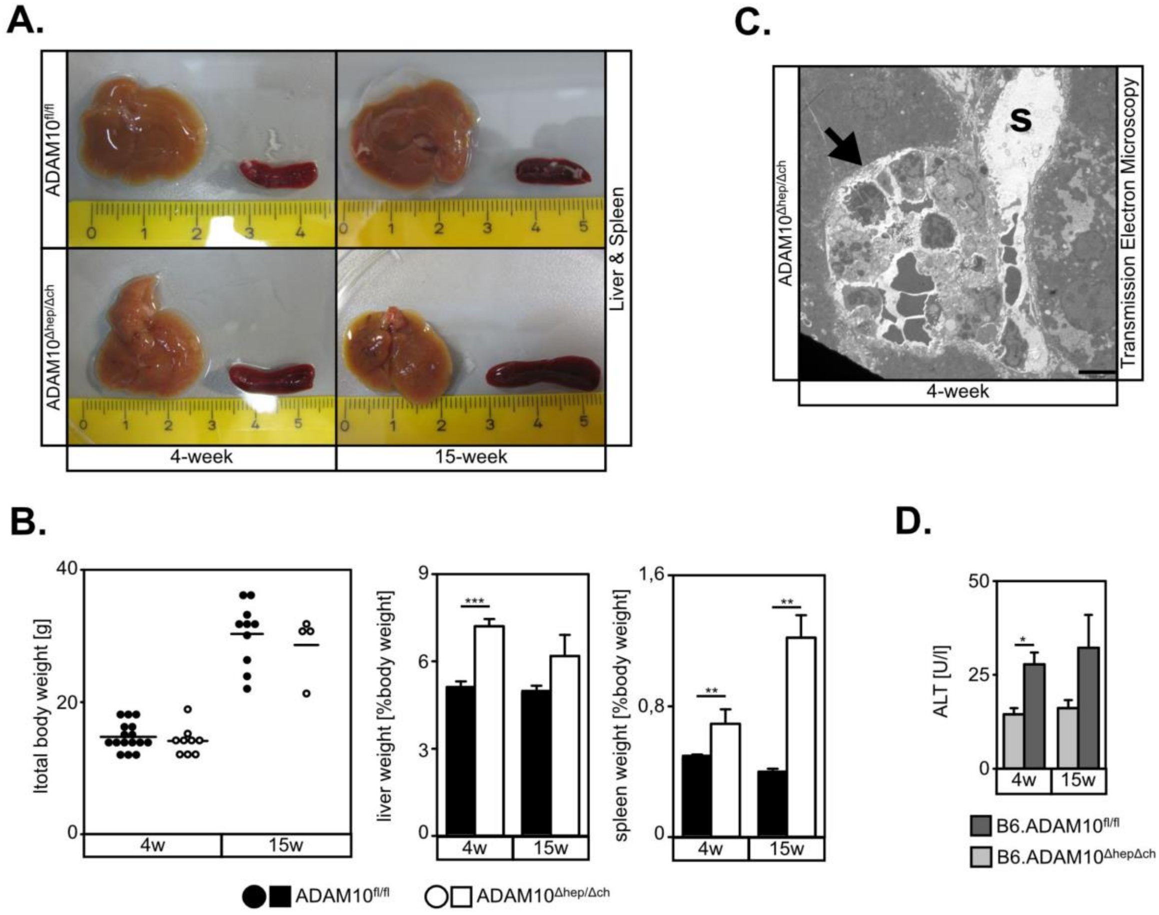

Supplementary Figure 2. A.+B. ADAM10$^{\Delta hep/\Delta ch}$ mice develop hepatosplenomegaly. (n=4-16 animals per subgroup) **C.** Transmission electron micrograph of a necrotic hepatocyte marked by black arrow. S: sinusoid. Scale bar indicates 5 µm. **D.** ALT serum levels are slightly but significantly upregulated in B6.ADAM10$^{\Delta Hep/\Delta Ch}$ mice, indicating slower onset of disease in mice with a pure C57BL/6 background.

Data represent the mean ± standard error of the mean (*P<0.05; ** P<0,01; *** P<0,001).

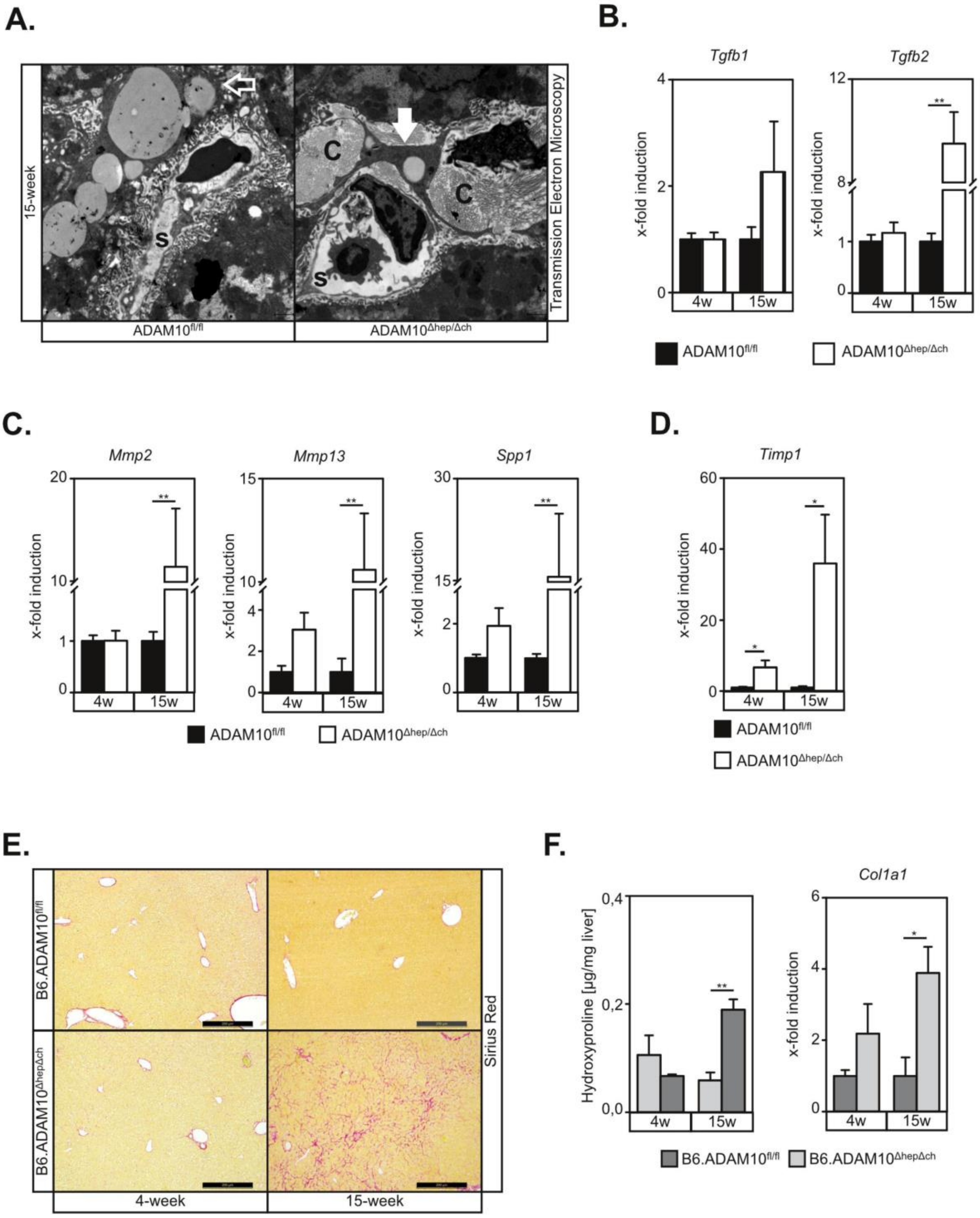
A.
15-week
s
C
C
s
Transmission Electron Microscopy
ADAM10fl/fl
ADAM10Δhep/Δch
B.
Tgfb1
Tgfb2
x-fold induction
4w
15w
ADAM10fl/fl
ADAM10Δhep/Δch
C.
Mmp2
Mmp13
Spp1
x-fold induction
4w
15w
ADAM10fl/fl
ADAM10Δhep/Δch
D.
Timp1
x-fold induction
4w
15w
ADAM10fl/fl
ADAM10Δhep/Δch
E.
B6.ADAM10fl/fl
B6.ADAM10Δhep/Δch
Sirius Red
4-week
15-week
F.
Col1a1
Hydroxyproline [µg/mg liver]
x-fold induction
4w
15w
B6.ADAM10fl/fl
B6.ADAM10Δhep/Δch

Supplementary Figure 3. A. Electronmicrographs of liver tissue sections reveal activated hepatic stellate cells (HSCs) in ADAM10$^{\Delta hep/\Delta ch}$ mice, as observed by a reduction of intracellular lipid vesicles and deposited collagen. Open arrow indicates quiescent HSC; filled arrow indicates activated HSC; C: collagen fibrils; S: sinusoid **B.** Expression of transforming growth factor beta 1 (*Tgfb1*) and 2 (*Tgfb2*) is upregulated in livers of ADAM10$^{\Delta hep/\Delta ch}$ mice as assessed by qPCR on mRNA from total liver tissue. (n=4-9 animals per subgroup) **C.** Expression of fibrosis-associated matrix metalloproteinases 2 (*MMP2*) and 13 (*MMP13*) and secreted phosphoprotein 1 (*Spp1*) is upregulated in livers of ADAM10$^{\Delta hep/\Delta ch}$ mice as assessed by qPCR on mRNA from total liver tissue. (n=4-9 animals per subgroup) **D.** Expression of fibrosis-associated *Timp1* is upregulated in livers of ADAM10$^{\Delta hep/\Delta ch}$ mice as assessed by qRT-PCR on mRNA from total liver, suggesting that activity of MMPs is counterbalanced. (n=4-5 animals per subgroup) **E.** B6.ADAM10$^{\Delta hep/\Delta ch}$ mice using Alb-Cre on a pure C57BL/6 background displayed slower onset of liver fibrosis. Sirius Red staining of histological liver sections shows collagen deposition in livers of 15-week old B6.ADAM10$^{\Delta Hep/\Delta Ch}$ mice. Scale bars indicate 200 µm. **F.** Quantification of hydroxyproline (n=2-4 animals per subgroup) content and analysis of *Col1A1* expression (n=3-4 animals per subgroup) in total liver extracts confirms mild liver fibrosis in B6.ADAM10$^{\Delta Hep/\Delta Ch}$ mice.

Data represent the mean ± standard error of the mean (*P<0.05; ** P<0,01).

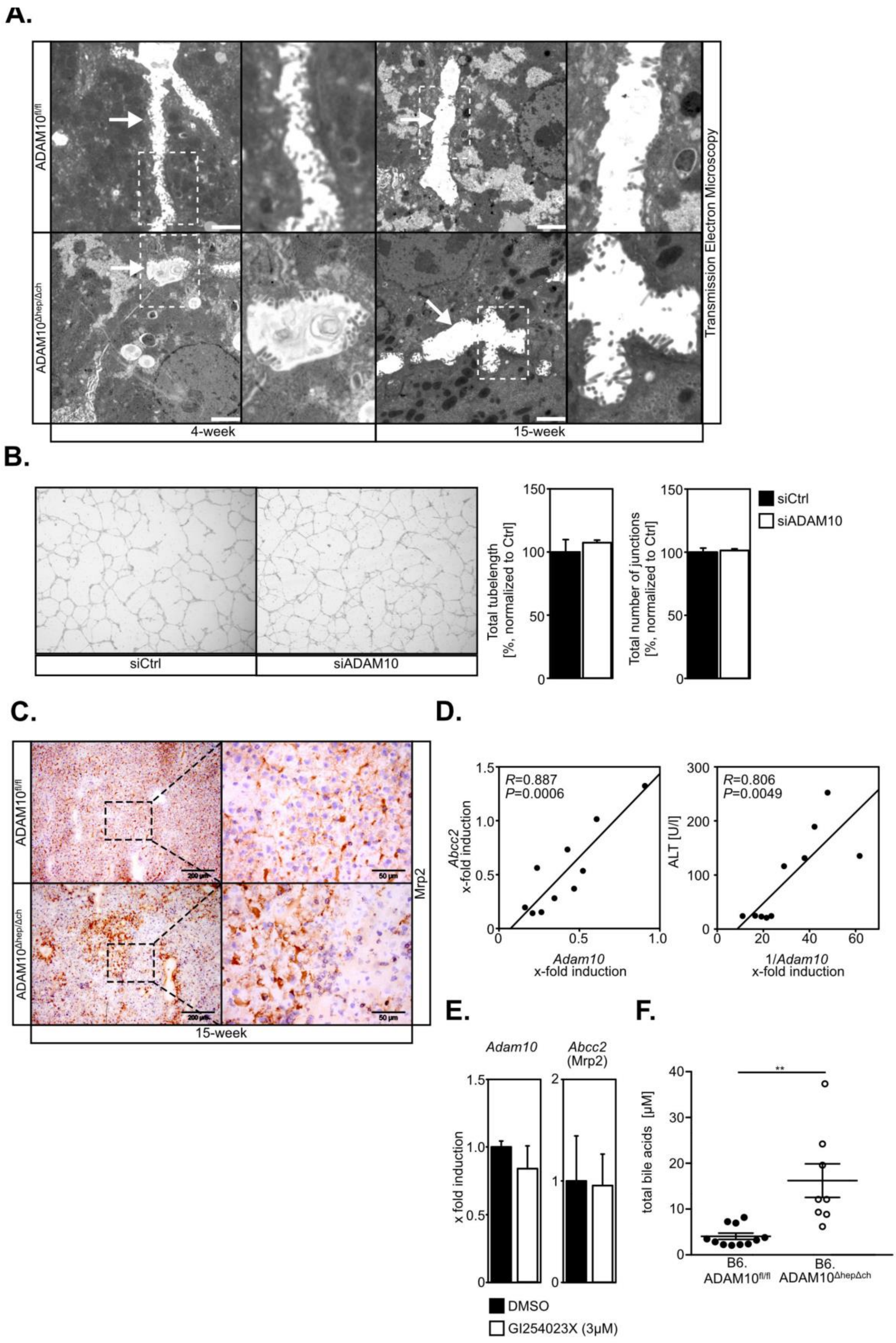

Supplementary Figure 4. A. Transmission electron microscopy of liver tissue sections displays no alterations in bile canaliculi morphology. Arrows mark bile canaliculi. Scale

bars indicate 1 µm. **B.** siRNA-mediated suppression of ADAM10 does not affect tube. formation of BMOL cells cultured on Matrigel, a model to study biliary tubulogenesis. Shown is one representative and the quantification of two independent experiments (n=2). **C.** Immunohistological staining of the bile acid transporter Mrp2 in liver tissue sections reveals reduction in Mrp2 in bile canaliculi of ADAM10-deficient livers. Scale bars indicate 200 µm and in magnified areas 50 µm. **D.** *Adam10* expression significantly correlates with *Abcc2* expression. Adam10 expression also significantly inversely correlates with serum ALT levels. (n=10 animals analysed) **E.** GI254023X-mediated inhibition of ADAM10 for 72 hours in the murine LPC line BMOL does not result in transcriptional repression of the *Abcc2* gene encoding for Mrp2. (n=3) **F.** Serum levels of total biliary acids are significantly increased in 30-32-week old B6.ADAM10$^{\Delta Hep/\Delta Ch}$ mice (n=8-11 animals per subgroup). Data represent the mean ± standard error of the mean (** P<0,01).

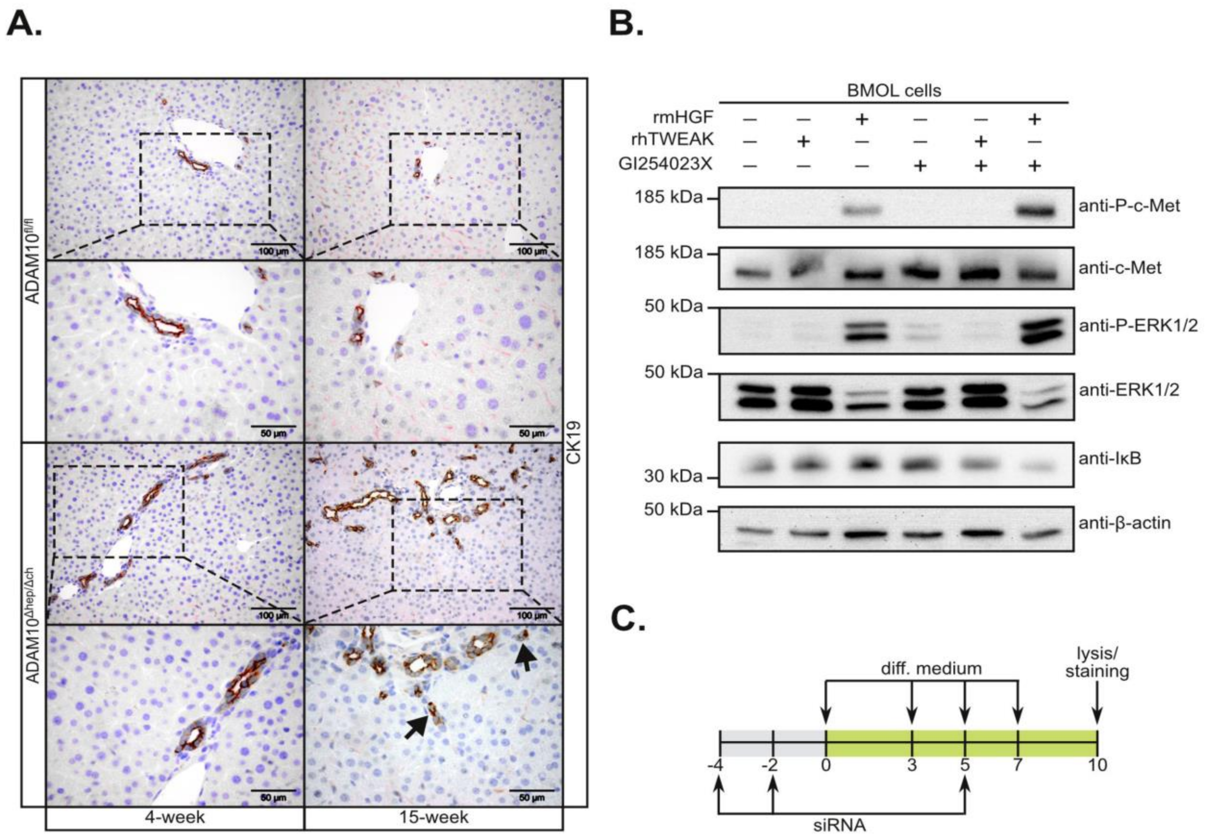

Supplementary Figure 5. A. Immunohistological staining of CK19+ liver progenitor and biliary cells demonstrates a ductular reaction in ADAM10-deficient livers. Black arrows indicate strings of proliferating liver progenitor cells in the liver parenchyma. Scale bars indicate 100 µm and in magnified areas 50 µm. **B.** HGF-induced c-Met signalling but not TWEAK-induced Fn14 signalling is increased in the presence of the ADAM10 inhibitor GI254023X. Shown is one out of three independent experiments. **C.** Schematic outline of BMOL differentiation experiment.

3. Supplementary Tables

Supplementary Table 1: Oligonucleotide Sequences for Genotyping and qRT-PCR

Targetgene	Forward Primer	Reverse Primer	Sequence Reference
Adam10 (gDNA)	5'-TACAACCATGCCCAGCTTTTTAGT-3'	5'-GCCGATGTGCCAGATGAGTG-3'	NC_000075
Hbbt1	5'-CCAATCTGCTCACACAGGATAGAGAGGGCAGG-3'	5'-CCTTGAGGCTGTCCAAGTGATTCAGGCCATCG-3'	NC_000073
Adam10	5'-GGGAAGAAATGCAAGCTGAA-3'	5'-CTGTACAGCAGGGTCCTTGAC-3'	NM_007399
Tnfsf12	5'-CAGGATGGAGCACAAGCAG-3'	5'-GGCTGGAGCTGTTGATTTTG-3'	NM_011614.3
Tnfrsf12a	5'-ATTCGGCTTGGTGTTGATG-3'	5'-CCATGCACTTGTCGAGGTC-3'	NM_013749.2
Acta2	5'-GCATCCACGAAACCACCTAT-3'	5'-AGGTAGACAGCGAAGCCAAG-3'	X13297
Col1a1	5'-GAGCGGAGAGTACTGGATCG-3'	5'-TACTCGAACGGGAATCCATC-3'	NM_007742
Spp1	5'-CTCTGATCAGGACAACAAC-3'	5'-CCTCAGAAGATGAACTCTC-3'	AF515708
Tgfb2	5'-CCTTCGCCCTCTTTACATTG-3'	5'-TTCGATCTTGGGCGTATTTC-3'	NM_009367
Tgfb1	5'-TGGAGCAACATGTGGAACTC-3'	5'-GTCAGCAGCCGGTTACCA-3'	NM_011577
Mmp2	5'-CAGCAAGTAGATGCTGCC-3'	5'-CAGCAGCCAGCCAGTC-3'	NM_008610
Mmp13	5'-TTTGAGAACACGGGGAAGAC-3'	5'-TGGGCCCATTGAAAAAGTAG-3'	NM_008607
Timp1	5'-GCAAAGAGCTTTCTCAAAGACC-3'	5'-AGGGATAGATAAACAGGGAAACACT-3'	NM_001044384.1/NM_011593.2/NM_001294280.2
Abcc2	5'-CAAATCCAATTCTCTACCTATGCAC-3'	5'-GCCTGCAGTGTTGGATCA-3'	NM_013806.2
Abcc3	5'-TCCGAAACTACGCACCAGAT-3'	5'-CTCATTTGCATTTTGCAAGG-3'	NM_029600.3
Abcb11	5'-AGTGGCTGCTTTTGGTGGT-3'	5'-CAGCGCTGAGCAAACATAAG-3'	NM_021022.3
Slco1b2	5'-CCCGTGACTAATCCAACAACA-3'	5'-GCTTCTCAGAGACCATAGAAAACC-3'	NM_020495.1
Abcb4	5'-TTGAACTAGGCAGCATCAGC-3'	5'-AACAGTGTCAACAGGCCAATTA-3'	NM_008830.2
Tuba1a1	5'-CTGGAACCCACGGTCATC-3'	5'-GTGGCCACGAGCATAGTTATT-3'	NM_011653
Actb	5'-TGGAATCCTGTGGCATCCATGAAA-3'	5'-TAAAACGCAGCTCAGTAACAGTCCG-3'	NM_007393
Alb	5'- TGACCCAGTGTTGTGCAGAG-3'	5'- TTCTCCTTCACACCATCAAGC-3'	NM_009654.3
HNF4a	5'- CAGCAATGGACAGATGTGTGA-3'	5'- TGGTGATGGCTGTGGAGTC-3'	NM_008261.2
HNF6	5'- AGACCTTCCGGAGGATGTG-3'	5'- TTGCTCTTTCCGTTTGCAG-3'	BC024053.1
HNF1b	5'- GACACTCCTCCCATCCTCAA-3'	5'- CATGTATCCCTTGATCATTTTGG-3'	X55842.1

Supplementary Table 2: Antibodies

Target	Host species	Dilution	Distibuted by
**Immunoblotting**			
ADAM10 (GTX63486)	rabbit	1:1000	Genetex
β-actin (A2066)	rabbit	1:10000	Sigma-Aldrich, Steinheim, Germany
GFP (7.1/13.1)	rabbit	1:1000	Roche, Penzberg, Germany
P-c-Met (Y1234/1235) (D26)	rabbit	1:1000	Cell signaling/NEB, Frankfurt a. M., Germany
c-Met (25H2)	mouse	1:1000	Cell signaling/NEB, Frankfurt a. M., Germany
P-Erk1/2	rabbit	1:1000	Cell signaling/NEB, Frankfurt a. M., Germany
Erk1/2	mouse	1:1000	Cell signaling/NEB, Frankfurt a. M., Germany
IκB	mouse	1:1000	Cell signaling/NEB, Frankfurt a. M., Germany
CK19 (TromaIII)	rat	1:1000	DSHB, Iowa City, USA
HNF1β (sc22840)	rabbit	1:1000	Santa Cruz/BioTechne, Heidelberg, Germany
HNF4α (sc6556)	goat	1:1000	Santa Cruz/BioTechne, Heidelberg, Germany
E-cadherin (#3195)	rabbit	1:1000	Cell signaling/NEB, Frankfurt a. M., Germany
β-catenin	mouse	1:1000	BD Bioscience, Heidelberg, Germany
**Immunohistochemistry/Immunfluorescence**			
CK19 (TromaIII)	rat	1:500 (IHC)<br>1:200 (IF)	DSHB, Iowa City, USA
F4/80	rat	1:150	Ruth Ganss, Perth, Australia
Ki67	rabbit	1:200	Pierce Thermo Scientific, Bonn, Germany and<br>Cell signalling/Genesearch, Arundel, Australia
CD44 (IM7)	rat	1:150	eBiosciences, Frankfurt a.M., Germany
panCK	rabbit	1:400	Dako, North Sidney, Australia
αSMA (1A4)	mouse	1:400	Dako, Hamburg, Germany
GFP	rabbit	1:1000	abcam
Mrp2 (M2III-6)	mouse	1:200	Biozol, Eching, Germany
HNF1β (sc22840)	rabbit	1:100	Santa Cruz/BioTechne, Heidelberg, Germany
HNF4α (sc6556)	goat	1:100	Santa Cruz/BioTechne, Heidelberg, Germany
HNF6 (sc	rabbit	1:100	Santa Cruz/BioTechne, Heidelberg, Germany
E-cadherin (#3195)	rabbit	1:200	Cell signaling/NEB, Frankfurt a. M., Germany
β-catenin	mouse	1:100	BD Bioscience, Heidelberg, Germany

References

Sparks, E.E., Perrien, D.S., Huppert, K.A., Peterson, T.E. and Huppert, S.S. (2011). Defects in hepatic Notch signaling result in disruption of the communicating intrahepatic bile duct network in mice. Dis Model Mech *4*:359-367.
Tirnitz-Parker, J.E.E., Tonkin, J.N., Knight, B., Olynyk, J.K. and Yeoh, G.C.T. (2007). Isolation, culture and immortalisation of hepatic oval cells from adult mice fed a choline-deficient, ethionine-supplemented diet. Int J Biochem Cell Biol *39*:2226-2239.
Uchinami, H., Seki, E., Brenner, D.A. and D'Armiento, J. (2006). Loss of MMP 13 attenuates murine hepatic injury and fibrosis during cholestasis. Hepatology *44*:420-429.
